# Behavioral effects of multiple-dose oxytocin treatment in autism: a randomized, placebo-controlled trial with long-term follow-up

**DOI:** 10.1186/s13229-020-0313-1

**Published:** 2020-01-15

**Authors:** Sylvie Bernaerts, Bart Boets, Guy Bosmans, Jean Steyaert, Kaat Alaerts

**Affiliations:** 1 Department of Rehabilitation Sciences, Research Group for Neurorehabilitation, KU Leuven, Tervuursevest 101 box 1501, 3001 Leuven, Belgium; 2 Leuven Autism Research consortium, KU Leuven, Leuven, Belgium; 3 Department of Neurosciences, Center for Developmental Psychiatry, KU Leuven, Kapucijnenvoer 7 blok h - box 7001, 3000 Leuven, Belgium; 4 Faculty of Psychology and Educational Sciences, Parenting and Special Education Research Group, KU Leuven, Leopold Vanderkelenstraat 32 box 3765, 3000 Leuven, Belgium; 5 Department of Neurosciences, Center for Developmental Psychiatry, KU Leuven, O&N II Herestraat 49 box 7003, 3000 Leuven, Belgium

**Keywords:** Autism spectrum disorder, Oxytocin, Repetitive and restricted behavior, Social responsiveness, Attachment

## Abstract

**Background:**

Intranasal administration of the “prosocial” neuropeptide oxytocin is increasingly explored as a potential treatment for targeting the core characteristics of autism spectrum disorder (ASD). However, long-term follow-up studies, evaluating the possibility of long-lasting retention effects, are currently lacking.

**Methods:**

Using a double-blind, randomized, placebo-controlled, parallel design, this pilot clinical trial explored the possibility of long-lasting behavioral effects of 4 weeks of intranasal oxytocin treatment (24 International Units once daily in the morning) in 40 adult men with ASD. To do so, self-report and informant-based questionnaires assessing core autism symptoms and characterizations of attachment were administered at baseline, immediately after 4 weeks of treatment (approximately 24 h after the last nasal spray administration), and at two follow-up sessions, 4 weeks and 1 year post-treatment.

**Results:**

No treatment-specific effects were identified in the primary outcome assessing social symptoms (Social Responsiveness Scale, self- and informant-rated). In particular, with respect to self-reported social responsiveness, improvements were evident both in the oxytocin and in the placebo group, yielding no significant between-group difference (*p* = .37). Also informant-rated improvements in social responsiveness were not significantly larger in the oxytocin, compared to the placebo group (between-group difference: *p* = .19). Among the secondary outcome measures, treatment-specific improvements were identified in the Repetitive Behavior Scale and State Adult Attachment Measure, indicating reductions in self-reported repetitive behaviors (*p* = .04) and reduced feelings of avoidance toward others (*p* = .03) in the oxytocin group compared to the placebo group, up to 1 month and even 1 year post-treatment. Treatment-specific effects were also revealed in screenings of mood states (Profile of Mood States), indicating higher reports of “vigor” (feeling energetic, active, lively) in the oxytocin, compared to the placebo group (*p* = .03).

**Conclusions:**

While no treatment-specific improvements were evident in terms of core social symptoms, the current observations of long-term beneficial effects on repetitive behaviors and feelings of avoidance are promising and suggestive of a therapeutic potential of oxytocin treatment for ASD. However, given the exploratory nature of this pilot study, future studies are warranted to evaluate the long-term effects of OT administration further.

**Trial registration:**

The trial was registered with the European Clinical Trial Registry (Eudract 2014-000586-45) on January 22, 2014 (https://www.clinicaltrialsregister.eu/ctr-search/trial/2014-000586-45/BE).

## Background

Autism spectrum disorder (ASD) is characterized by lifelong impairments in social and communicative functioning, and the presence of stereotyped behaviors and interests [1]. In the past decade, intranasal administration of the neuropeptide oxytocin (OT) has increasingly been explored as a potential pharmacological treatment for targeting the core characteristics of ASD. Endogenous OT is synthesized in the hypothalamus where neurons of the paraventricular nuclei project to various areas of the central nervous system involved in complex (social) behaviors (e.g., amygdala). In typically developing individuals, OT has been linked to interpersonal bonding, parental care, and the ability to establish trust and form social attachments [2, 3].

With respect to ASD, initial clinical trials have demonstrated that a single dose of OT can induce behavioral enhancements on tasks assessing repetitive behavior [4], affective speech comprehension (emotional intonations) [5], facial emotion recognition [6], and social decision making (cyberball computer game) [7]. Multiple-dose trials assessing the effects of OT treatment in adults with ASD have also shown beneficial effects. Anagnostou et al. [8] assessed the safety and efficacy of 6 weeks of intranasal OT treatment on core autism symptom domains (social cognition/function and repetitive behaviors) in 19 adults with ASD (16 men, three women), and showed improved emotion recognition, quality of life, and tentative improvements in repetitive behaviors after OT treatment. In another study with 20 adult men with ASD, Watanabe et al. [9] studied the effects of 6 weeks of intranasal OT administration on core autism characteristics and showed significant improvements in social reciprocity and social functioning (social-judgment task). In a more recent large-scale trial, Yamasue et al. [10] also assessed the effects of 6 weeks of intranasal OT treatment on core autism symptom domains in 106 adult men with ASD and showed significant improvements in repetitive behaviors.

However, a more mixed pattern of results emerged from studies assessing the effects of multiple-dose OT treatment in children with ASD. For example, Dadds et al. [11] assessed behavioral effects of a 4-day intranasal OT treatment in 38 boys with ASD (7 to 16 years old) during parent-child interaction training, but found no OT-specific improvements in repetitive behaviors or social responsiveness. Later, also Guastella et al. [12] failed to show beneficial effects after an 8-week OT treatment on core ASD characteristics in slightly older boys (12 to 18 years old). On the other hand, a more recent trial assessing the effects of 5 weeks of intranasal OT treatment on core ASD characteristics in 31 children with ASD (27 boys, four girls) was able to demonstrate improved social responsiveness as reported by the parents [13]. Similarly, Parker et al. [14] investigated whether a 4-week intranasal OT treatment could improve core autism characteristics in 32 children (6–12 years old) with ASD and showed an increase in parent-reported social responsiveness. Taken together, findings of these initial multiple-dose trials provided indications for a more consistent (positive) pattern of results for OT trials with adults with ASD [8–10], compared to trials with children with ASD [11–14].

To date, however, potential long-term effects of OT treatment that outlast the period of actual administration have not yet been addressed in adults with ASD. These assessments, however, would be of high relevance since repeated administrations over an extended period might induce long-lasting, experience-dependent adaptations within neural circuits. With respect to ASD, it has been postulated that early-life impairments in social attention/orienting may deprive patients of adequate social learning experiences that normally drive the typical development of social brain networks [15]. Considering that OT is hypothesized to increase the saliency of social cues [16], OT therapy might induce an enrichment of social experiences that stimulates long-term adaptations in social behaviors.

In this pilot study, we assessed—for the first time—the possibility of long-term retention effects of 4 weeks of intranasal OT administration on core autism symptom domains (including social responsiveness and restricted and repetitive behaviors), attachment characteristics, and general aspects of quality of life in adult men with ASD. While prior multiple-dose trials mainly explored the effects of treatment on these core autism symptom domains, a recent study from our lab [17] showed that 2 weeks of OT treatment in typically developing young-adult men reduced self-reported feelings of attachment avoidance and increased self-reported feelings of secure attachment toward peers. Against this background and given the growing body of research demonstrating an important role of the oxytocinergic system in interpersonal trust and attachment [18–20], the current study also included an initial assessment of attachment measures to evaluate the long-term effects of OT treatment in adults with ASD. With respect to ASD, Rutgers et al. [21] suggested that most children with ASD (53%) are able to form a secure attachment to caregivers, but that they are significantly less likely to do so compared to typically developing children. In addition, a recent meta-analysis concluded that more severe autism characteristics are associated with less secure attachment in children with ASD [22].

The effects of treatment on core autism characteristics and attachment were assessed immediately after the 4-week treatment period, and at follow-up sessions, 4 weeks and 1 year post-treatment, to assess the possibility of retention effects that outlast the period of actual administration. In line with prior multiple-dose studies [8, 9, 17], we hypothesized that multiple doses of OT would improve self-report and informant-based ratings of social responsiveness and repetitive behaviors, and ameliorate self-ratings of attachment characteristics and quality of life. A key question was to evaluate whether any beneficial effects of multiple-dose OT treatment would outlast the period of actual administration until 1 month (4 weeks) and/or 1 year post-treatment.

## Materials and Methods

### General study design

**Fig. 1:**
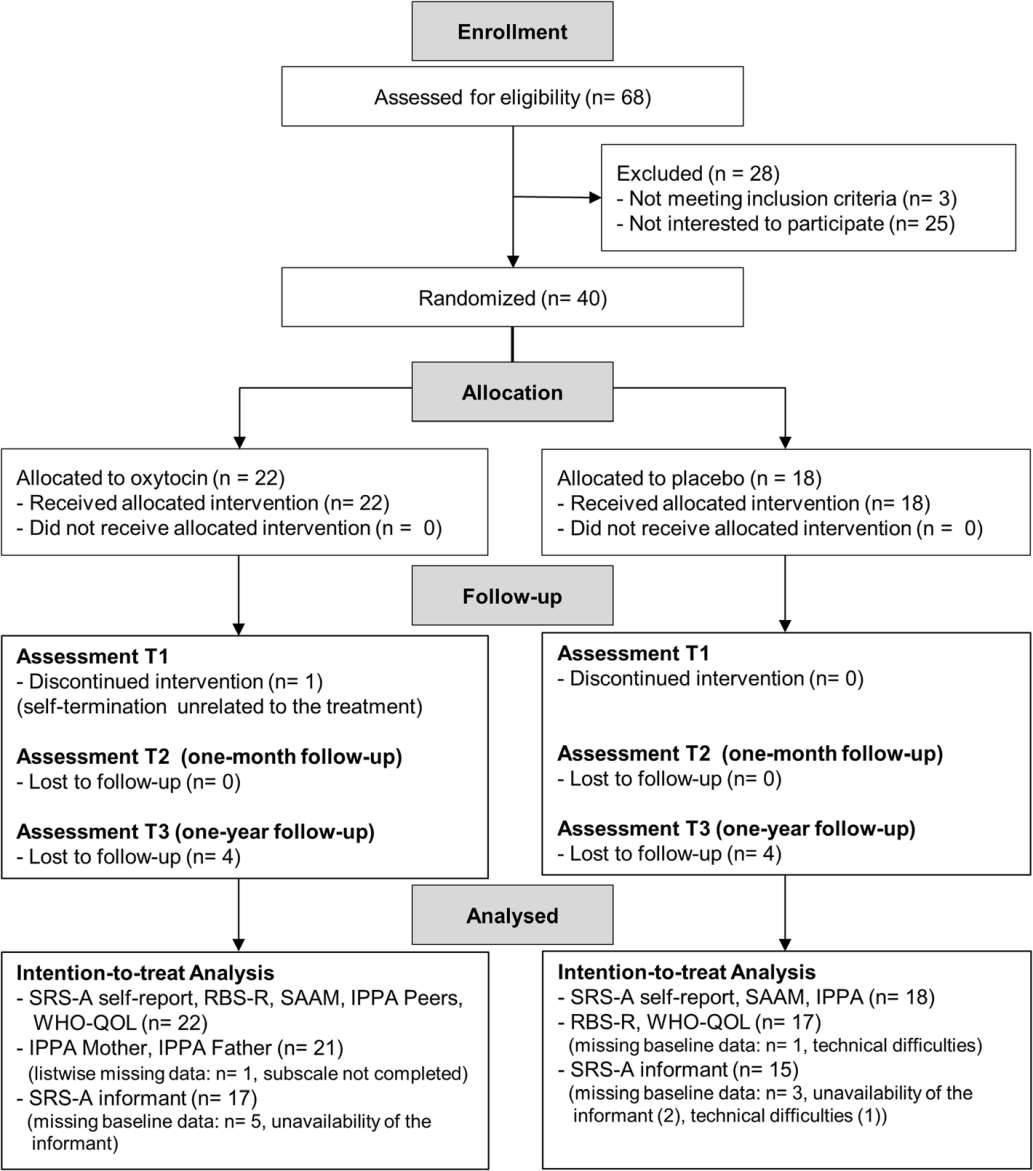
CONSORT Flow diagram. Data were analyzed using an intention-to-treat format with last-observations-carried-forward to replace missing data. For participants with missing baseline data, analysis for that measure was excluded list-wise. *SRS*-*A* Social Responsiveness Scale adult version, *RBS*-*R* Repetitive Behavior Scale Revised, *SAAM*: State Adult Attachment Measure, *IPPA* Inventory of Parent and Peer Attachment, *WHO*-*QOL* World Health Organization Quality of Life

This two-arm, double-blind, randomized, placebo-controlled parallel study was performed at the Leuven University Hospital (Leuven, Belgium) to assess multiple-dose effects of intranasal oxytocin (OT) administration on core autism characteristics and experience of attachment in adult men with ASD. A specific aim of this pilot clinical trial was to assess whether any treatment-induced effects would outlast the period of actual administration. To do so, changes-from-baseline (T0) in self-report and informant-based questionnaire scores were assessed immediately after 4 weeks of OT treatment (T1), and at two follow-up sessions, 4 weeks (T2) and 1 year post-treatment (T3) (see Fig. 1, CONSORT Flow diagram for number of participants randomized and analyzed). Written informed consent was obtained from all participants prior to the study. Consent forms and study design were approved by the local Ethics Committee for Biomedical Research at the University of Leuven, KU Leuven (S56327) in accordance to The Code of Ethics of the World Medical Association (Declaration of Helsinki). The trial was registered with the European Clinical Trial Registry (Eudract 2014-000586-45) and the Belgian Federal Agency for Medicines and Health products. Note that the data presented in the current report are part of a larger clinical trial in which neural measures (i.e., task-based and resting-state magnetic resonance imaging (MRI)) were assessed in addition to the presented questionnaire data. These MRI modalities are however not part of the current report and will be reported elsewhere (manuscripts in preparation).

### Participants

Forty high-functioning adult men with a formal diagnosis of ASD were recruited between April 2015 and December 2016 from the Autism Expertise Centre at the Leuven University Hospital. The diagnosis was established by a multidisciplinary team (child psychiatrist and/or expert neuropediatrician, psychologist, speech/language pathologist, and/or physiotherapist) based on the strict criteria of the DSM-IV-TR (Diagnostic and Statistical Manual of Mental Disorders) [1]. Prior to the intervention, the Autism Diagnostic Observation Schedule (ADOS or ADOS-2) [23, 24] and estimates of intelligence (6-subtest short version of the Wechsler Adult Intelligence Scale-IV Dutch version: Block design, Digit span, Similarities, Vocabulary, Symbol search and Visual puzzles) [25] were acquired from all participants (Table 1). Inclusion criteria comprised a clinical diagnosis of autism spectrum disorder; gender (male); and age (18–35 years old). Exclusion criteria for participation comprised any neurological disorder (e.g., stroke, epilepsy, concussion); demonstrated genetic disorder; or any contraindication for MRI (note that the MRI data analyses are not part of the current report). Current psychoactive medication use and the presence of comorbid psychiatric disorders were screened (Additional file 1: Table S1). Forty participants were randomly allocated to either the OT (*n* = 22) or the placebo (PL) group (*n* = 18) (see Fig. 1, CONSORT Flow diagram). The initial sample size (*n* = 40) was set to be comparable to three prior studies showing significant effects of multiple-dose OT treatment on similar outcome measures [8, 9, 17].

**Table 1:** Participant characteristics

	Oxytocin	Placebo	*T* value	*p* value
Number of participants	22	18		
Age	25.00 ± 4.86	24.00 ± 5.55	0.62	0.54
IQ				
Total IQ	102.27 ± 12.45	104.61 ± 21.59	-0.43	0.67
VIQ	105.57 ± 9.27	108.72 ± 16.83	-0.74	0.47
PIQ	104.76 ± 18.35	102.39 ± 22.90	0.36	0.72
ADOS(2)				
Total	7.18 ± 4.22	8.06 ± 4.26	-0.65	0.52
Communication	2.05 ± 1.40	2.39 ± 1.54	-0.74	0.46
Social interaction	4.82 ± 3.50	5.67 ± 3.33	-0.78	0.44
Use of psychostimulant medication	6	2		
Comorbidity	8	2		

Data are shown as mean ± standard deviation. *IQ* intelligence quotient, *VIQ* verbal IQ, *PIQ* performance IQ, *ADOS*(2) Autism Diagnostic Observation Schedule(2). Detailed information on medication use and comorbidities is provided in Additional file 1: Table S1. Note that for one participant of the OT group only total IQ information was available, but not VIQ or PIQ, so that the mean (± standard deviation) information of the VIQ and PIQ of the OT group are based on data from 21 participants

### Intervention

Participants were assigned to receive the OT or PL treatment based on a computer-generated randomized order. Except for the manager of randomization, all research staff conducting the trial, participants, and their parents and/or partners were blinded to treatment allocation. OT (Syntocinon®, Sigma-tau) and PL (saline natrium-chloride solution) were administered in amber 15 ml glass bottles with metered pump (ACA Pharma). Each puff per nostril contained four international units (IU) of OT. Participants self-administered a daily dose of 24 IU (three puffs/nostril) over four consecutive weeks (28 doses in total). This dose is in accordance with prior OT administration studies in neurotypical (young) adults [26, 27] and adults and adolescents with ASD [6, 28, 29]. At this dosage, no side effects or contraindications of OT have been described [29]. All participants received clear instructions about the use of the nasal spray [17, 30] and were monitored onsite until approximately 2 h after first nasal spray administration. During the course of the treatment, participants were asked to administer the nasal spray in the morning, to keep a daily record of the time point of nasal spray administration, and whether or not they were alone or in company of others the first 2 h after administration. Percentage of days at which the spray was administered in the presence of others was not significantly different between treatment groups (OT: 36.8 % (SD 29.9); PL: 36.0 % (SD 25.1); *t*(37) = .09; *p* = .93). Participants administered the nasal spray (OT or PL) daily during four consecutive weeks and at the end of each week participants were screened for potential adverse events, side effects (Additional file 1: Table S2), or changes in mood with the Profile of Mood States (POMS) questionnaire (Table 2 and Additional file 1: Figure S1). Finally, at the end of the trial, participants were asked if they thought they had received OT or PL. The majority of participants thought they had received the PL treatment (79.5%). The proportion of participants that believed they had received the OT treatment was not significantly larger in the actual OT group (28.57%), compared to the PL group (11.11%) (*p* = .18). Nonetheless, secondary analyses were performed to explore whether treatment effects were modulated by the participants’ own belief about the received treatment (Additional file 1: Supplementary Results).

**Table 2:** Outcome measures and effects of oxytocin treatment. Mean pre-to-post change scores are listed separately for each treatment group (oxytocin, placebo) and assessment session (T1, T2, T3). Cohen’s d effect sizes of *between*-*group* differences are reported separately for each outcome measure and assessment session. *T* and *p* values correspond to single-sample *t* tests assessing *within*-*group* changes from baseline separately for the oxytocin and placebo group

Outcome measure	Oxytocin				Placebo				Between-group difference
	*N*	Mean ± SD	*T* value	*p*	*N*	Mean ± SD	*T* value	p	Cohen’s d
Multiple-dose effect (T1)									
Primary outcomes									
SRS-A self-report	22	− 5.55 ± 11.40	− 2.28	0.033	18	− 1.06 ± 10.01	− 0.45	0.66	−0.42
SRS-A informant-based	17	0.0 ± 15.86	0.00	1.00	15	− 0.87 ± 12.83	− 0.26	0.80	0.10
Secondary outcomes									
RBS-R	22	− 4.77 ± 6.47	− 3.46	0.002	17	− 1.76 ± 4.75	− 1.53	0.15	− 0.63
SAAM avoidance	22	− 0.40 ± 0.71	− 2.63	0.016	18	0.06 ± 0.98	0.24	0.81	− 0.61
SAAM security	22	0.27 ± 0.77	1.62	0.12	18	− 0.05 ± 0.66	− 0.31	0.76	0.63
SAAM anxiety	22	− 0.14 ± 0.75	− 0.90	0.38	18	0.28 ± 0.95	1.24	0.23	− 0.62
IPPA Peers	22	1.45 ± 3.85	1.77	0.091	18	0.56 ± 4.05	0.58	0.57	0.30
IPPA Mother	21	− 0.52 ± 2.71	− 0.88	0.39	18	0.44 ± 3.45	0.55	0.59	− 0.39
IPPA Father	21	0.43 ± 3.30	0.60	0.56	18	− 0.61 ± 3.81	− 0.68	0.50	0.29
WHO-QOL	22	1.77 ± 8.04	1.03	0.31	17	− 1.35 ± 6.74	− 0.83	0.42	0.63
Profile of mood states									
Tension	22	− 2.00 ± 2.29	− 4.10	0.0005	18	− 2.39 ± 3.03	− 3.34	0.004	0.14
Anger	22	0.00 ± 4.05	0.00	1.00	18	− 0.61 ± 2.73	− 0.95	0.35	0.18
Depression	22	− 1.14 ± 4.50	− 1.19	0.25	18	− 0.33 ± 2.81	− 0.50	0.62	− 0.21
Vigor	22	− 1.00 ± 2.53	− 1.86	0.077	18	− 2.94 ± 3.64	− 3.43	0.003	0.62
Fatigue	22	− 2.09 ± 3.99	− 2.46	0.023	18	− 1.11 ± 5.12	− 0.92	0.37	− 0.21
One-month retention effect (T2)									
Primary outcomes									
SRS-A self-report	22	− 5.64 ± 12.57	− 2.10	0.048	18	− 7.67 ± 12.09	− 2.69	0.015	0.22
SRS-A informant-based	17	− 9.59 ± 10.98	− 3.60	0.002	15	− 1.20 ± 10.73	− 0.43	0.67	− 0.83
Secondary outcomes									
RBS-R	22	− 4.91 ± 6.33	− 3.64	0.002	17	− 2.35 ± 3.43	− 2.83	0.012	− 0.50
SAAM avoidance	22	− 0.38 ± 0.70	− 2.58	0.018	18	− 0.06 ± 0.76	− 0.35	0.73	− 0.53
SAAM security	22	0.04 ± 1.01	0.18	0.86	18	− 0.40 ± 0.99	− 1.70	0.11	0.62
SAAM anxiety	22	0.08 ± 1.05	0.36	0.72	18	0.11 ± 0.87	0.54	0.60	− 0.05
IPPA Peers	22	1.32 ± 3.71	1.67	0.11	18	0.06 ± 3.70	0.06	0.95	0.45
IPPA Mother	21	− 0.38 ± 3.43	− 0.51	0.62	18	0.06 ± 4.35	0.05	0.96	− 0.14
IPPA Father	21	0.52 ± 3.59	0.67	0.51	18	− 0.33 ± 3.87	− 0.37	0.72	0.31
WHO-QOL	22	1.14 ± 5.48	0.97	0.34	17	0.35 ± 4.53	0.32	0.75	0.24
Profile of mood states									
Tension	22	− 2.64 ± 2.80	− 4.41	0.0002	18	− 2.11 ± 3.22	− 2.76	0.013	− 0.17
Anger	22	0.36 ± 3.68	0.46	0.65	18	− 0.39 ± 3.91	− 0.42	0.68	0.20
Depression	22	− 0.82 ± 2.63	− 1.46	0.16	18	0.22 ± 3.41	0.28	0.79	− 0.34
Vigor	22	0.14 ± 3.58	0.18	0.86	18	− 1.44 ± 4.33	− 1.42	0.17	0.40
Fatigue	22	− 2.69 ± 2.71	− 4.63	0.0001	18	− 2.33 ± 4.47	− 2.21	0.04	− 0.09
One-year retention effect (T3)									
Primary outcomes									
SRS-A self-report	22	− 8.59 ± 20.95	− 1.92	0.07	18	− 6.72 ± 21.01	− 1.36	0.19	− 0.12
SRS-A informant-based	17	− 7.41 ± 19.26	− 1.59	0.13	15	− 4.13 ± 24.64	− 0.65	0.53	− 0.18
Secondary outcomes									
RBS-R	22	− 4.91 ± 9.46	− 2.43	0.02	17	− 0.41 ± 4.27	− 0.40	0.70	− 0.98
SAAM avoidance	22	− 0.52 ± 1.18	− 2.07	0.05	18	0.0 ± 0.75	0.00	1.00	− 0.80
SAAM security	22	0.20 ± 1.52	− 0.62	0.54	18	− 0.14 ± 0.66	− 0.91	0.37	− 0.05
SAAM anxiety	22	0.17 ± 0.94	0.83	0.42	18	0.11 ± 1.21	0.39	0.70	0.06
IPPA Peers	22	0.68 ± 6.26	0.51	0.61	18	1.28 ± 4.17	1.30	0.21	− 0.20
IPPA Mother	21	0.33 ± 3.91	0.39	0.70	18	1.50 ± 5.44	1.17	0.26	− 0.30
IPPA Father	21	0.57 ± 4.08	0.64	0.53	18	− 0.50 ± 4.53	− 0.47	0.65	0.33
WHO-QOL	22	1.14 ± 8.37	0.64	0.53	17	0.29 ± 4.21	0.29	0.78	0.27
Profile of mood states									
Tension	22	− 1.86 ± 2.29	− 3.81	0.001	18	− 2.28 ± 3.46	− 2.79	0.012	0.14
Anger	22	0.59 ± 3.69	0.75	0.46	18	0.06 ± 3.84	0.06	0.95	0.14
Depression	22	0.50 ± 2.63	0.89	0.38	18	− 0.28 ± 3.51	− 0.34	0.74	0.25
Vigor	22	1.14 ± 3.88	1.37	0.18	18	− 0.61 ± 3.27	− 0.79	0.43	0.50
Fatigue	22	− 0.23 ± 6.04	− 0.18	0.86	18	0.39 ± 4.73	0.35	0.73	− 0.11

*SRS*-*A* Social Responsiveness Scale adult version, *RBS*-*R* Repetitive Behavior Scale-Revised, *SAAM* State Adult Attachment Measure, *IPPA* Inventory of Parent and Peer Attachment, *WHO*-*QOL* World Health Organization Quality of Life questionnaire. Screening for mood changes are based on the Profile of Mood States (POMS) questionnaire. For SRS-A, RBS-R, SAAM avoidance, SAAM anxiety, and POMS tension, anger, depression and fatigue; negative scores indicate pre-to-post improvement. Cohen’s d effect sizes (change from baseline_OT_–change from baseline_PL_)/pooled SD) are reported where 0.2 is indicative of a small effect, 0.5 a medium effect and 0.8 a large effect. Data printed in bold show Cohen’s d effect sizes equal to or larger than .50 (medium-sized effect)

### Outcome Measures

The Social Responsiveness Scale (for adults) (SRS-A) total score was used as the primary outcome measure (self-report and informant-based versions). The other behavioral questionnaires were considered secondary: Repetitive Behavior Scale-Revised (RBS-R); State Adult Attachment Measure (SAAM); Inventory of Parent and Peer Attachment (IPPA); Quality of Life questionnaire of the World Health Organization (WHO-QOL) (all self-report). More detailed information on each of the adopted questionnaires is provided in Additional file 1: Supplementary Methods.

In short, the SRS-A (64 items) [31] comprises four subscales examining social communication, social awareness, social motivation and rigidity/repetitiveness, using a four-point Likert-scale. SRS-A raw total scores were adopted.

The RBS-R (43 items) [32] examines a heterogeneous set of repetitive behaviors including stereotypic behavior, self-injurious behavior, compulsive behavior, ritualistic behavior, sameness behavior and restricted interests behavior, using a four-point Likert-scale. RBS-R raw total scores were adopted.

The SAAM [33] comprises three subscales examining attachment security (e.g., “I feel like I have someone to rely on”) (seven items); attachment anxiety (e.g., “I feel a strong need to be unconditionally loved right now”) (seven items); and attachment avoidance (e.g., “If someone tried to get close to me, I would try to keep my distance”) (seven items) using a seven-point Likert-scale. SAAM raw subscale scores were adopted.

The IPPA [34] examines trait attachment to (i) mother (12 items), (ii) father (12 items), and (iii) peers (12 items), using a four-point Likert-scale. The IPPA assesses attachment security along three dimensions: degree of mutual trust, quality of communication, and extent of anger and alienation. IPPA raw subscale scores were adopted.

Finally, the abbreviated version of the WHO-QOL [35] assesses general quality of life related to physical health, psychological health, social relationships, and environment using a five-point Likert scale. WHO-QOL raw total scores were adopted.

All questionnaires were assessed at baseline (T0), immediately after four consecutive weeks of nasal spray administration (approximately 24 h after the last nasal spray administration) (T1), and at follow-up sessions, 4 weeks (T2) and 1 year post-treatment (T3). Given that this is an exploratory pilot study evaluating the possibility of long-term retention effects of OT treatment, no primary time point was pre-specified.

### Data analysis

For each questionnaire, baseline differences between groups were assessed using two-sample *t* tests. To assess *between*-*group differences*, pre-to-post difference scores were calculated for each assessment session (T1, T2, T3) and difference scores were subjected to a linear mixed-effects model (one-tailed) with the random factor “subject,” and the fixed factors “treatment” (OT, PL), “session” (T1, T2, T3) and “treatment × session” interaction. An intention-to-treat format and last-observations-carried-forward to replace missing data was adopted. For participants with missing baseline data, analysis for that measure was excluded list-wise (Fig. 1 CONSORT Flow diagram). Cohen’s d effect sizes of between-group differences (change from baseline_OT_–change from baseline_PL_)/pooled standard deviation) are reported in Table 2, separately for each outcome measure and assessment session, where 0.2 is indicative of a small effect, 0.5 a medium effect, and 0.8 a large effect [36]. Additionally, pre-to-post difference scores were subjected to single-sample *t* tests to assess within-group changes (compared to baseline) in the OT group and PL group separately (Table 2). All statistics were executed with Statistica 8 (StatSoft. Inc. Tulsa, USA). Given that this is an exploratory pilot study evaluating long-term effects of OT treatment, the significance level was set at *p* < .05 for all analyses, without correction for multiple comparisons.

## Results

No significant baseline differences were revealed between participants allocated to the OT or PL group for any of the questionnaires (see Additional file 1: Table S3) or in terms of participant characteristics (see Table 1).

### Primary outcome—Social Responsiveness Scale

#### Self-rated SRS-A

**Fig. 2:**
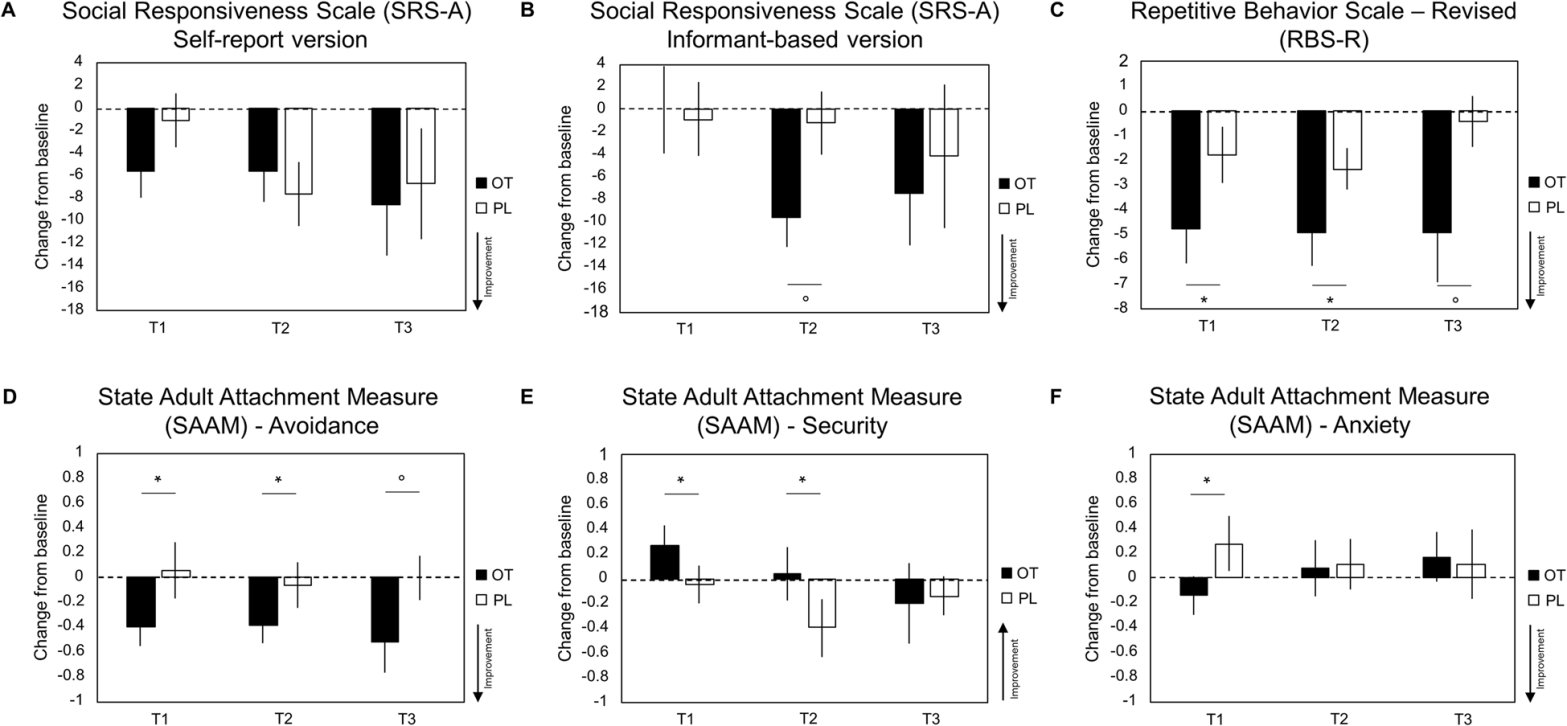
Effects of oxytocin treatment on autism symptoms and attachment. Mean pre-to-post changes (change from baseline) on self-report and informant-based questionnaires are visualized for the oxytocin (OT) and placebo (PL) treatment groups at assessment session “T1” (immediately after the four-week treatment), “T2” (at follow-up, one month post-treatment), and “T3” (at follow-up, 1 year post-treatment). Mean changes from baseline are visualized separately for **a** Social Responsiveness Scale (SRS-A) self-report version, **b** SRS-A informant-based version, **c** Repetitive Behavior Scale-Revised (RBS-R), **d** State Adult Attachment Measure (SAAM) Avoidance subscale, **e** SAAM Security subscale, and **f** SAAM Anxiety subscale. Lower scores indicate improvement for the SRS-A, RBS-R, SAAM Avoidance, and SAAM Anxiety questionnaires. For the SAAM Security questionnaire, higher scores indicate improvement. Vertical bars denote ± standard errors. Asterisks (*) indicate Cohen’s d ≥ .50 (medium-sized effect). Circles (°) indicate Cohen’s *d* ≥ .80 (large-sized effect)

Between-group analyses revealed no significant main effect of treatment (*F*(1,76) = .12, *p* = .37, ŋ^2^ = .00), nor a treatment × session interaction effect (*F*(2,76) = 1.17, *p* = .16, ŋ^2^ = .03), indicating that pre-to-post changes in self-reported social responsiveness were not significantly larger in the OT compared to the PL group (see Fig. 2 and Table 2 for the effect sizes of between-group differences separately for each session). Within-group analyses showed that Social Responsiveness Scale (SRS-A) scores were significantly reduced (compared to baseline) in the OT group at session T1 (immediately after treatment: *p* = .033), T2 (1 month post-treatment; *p* = .048), and at trend-level at session T3 (1 year post-treatment: *p* = .07). However, a similar reduction (compared to baseline) was evident in the PL group at session T2 (*p* = .015), indicating no specific benefit of OT over the PL treatment (see Table 2 reporting single-sample *t* tests assessing within-group changes from baseline).

#### Informant-rated SRS-A

Between-group analyses of the informant-rated SRS-A scores revealed no significant main effect of treatment (*F*(1, 60) = .78, *p* = .19, ŋ^2^ = .03), nor a treatment x session interaction effect (*F*(2, 60) = .83, *p* = .22, ŋ^2^ = .03), indicating that pre-to-post changes in informant-rated social responsiveness were not significantly larger in the OT compared to the PL group (see Fig. 2 and Table 2 for the effect sizes of between-group differences separately for each session). Note however that at session T2 (1 month post-treatment), informant-rated SRS-A scores were significantly reduced in the OT group (compared to baseline) (*p* = .002), but not in the PL group (*p* = .67) (see Table 2 reporting single-sample *t* tests assessing within-group changes from baseline).

### Secondary outcome—Repetitive Behavior Scale-Revised

In terms of repetitive behaviors, between-group analyses identified a significant main effect of treatment (*F*(1, 74) = 3.20, *p* = .04, ŋ^2^ = .08) (but no treatment x session interaction: *F*(2, 74) = 1.04, *p* = .18, ŋ^2^ = .03), indicating that across assessment sessions, pre-to-post improvements in repetitive behaviors were significantly larger in the OT compared to the PL group (see Fig. 2 and Table 2 for the effect sizes of between-group differences separately for each session). Within-group analyses confirmed that Repetitive Behavior Scale-Revised (RBS-R) scores were significantly reduced (compared to baseline) in the OT group at session T1 (immediately after treatment: *p* = .002), T2 (1 month post-treatment; *p* = .002), and session T3 (1 year post-treatment: *p* = .02), but not consistently in the PL group (T1: *p* = .15; T2: *p* = .012; T3: *p* = .70) (see Table 2 reporting single-sample *t* tests assessing within-group changes from baseline).

### Secondary outcome—State Adult Attachment Measure

#### Attachment avoidance

In terms of attachment avoidance, between-group analyses identified a significant main effect of treatment (*F*(1,76) = 3.70, *p* = .03, ŋ^2^ = .09) (but no treatment x session interaction: *F*(2,76) = .27, *p* = .38, ŋ^2^ = .01), indicating that across assessment sessions, pre-to-post improvements in attachment avoidance were significantly larger in the OT compared to the PL group (see Fig. 2 and Table 2 and for the effect sizes of between-group differences separately for each session). Within-group analyses confirmed that attachment avoidance scores were significantly reduced (compared to baseline) in the OT group at session T1 (immediately after treatment: *p* = .016), T2 (1-month post-treatment; *p* = .018), and session T3 (1-year post-treatment: *p* = .05), but not in the PL group (T1: *p* = .81; T2: *p* = .73; T3: *p* = 1.00) (see Table 2 reporting single-sample *t* tests assessing within-group changes from baseline).

#### Attachment security

Between-group analyses identified no main effect of treatment *F*(1,76) = .88, *p* = .18, ŋ^2^ = .02), nor a treatment × session interaction effect (*F*(2,76) = 1.08, *p* = .17, ŋ^2^ = .03), indicating no treatment-specific improvement in attachment security across assessment sessions (see Fig. 2 and Table 2 for the effect sizes of between-group differences separately for each session). Also no significant within-group pre-to-post changes were identified in the OT or PL group separately (see Table 2).

#### Attachment anxiety

Between-group analyses identified no main effect of treatment (*F*(1,76) = .25, *p* = .31, ŋ^2^ = .01), nor a treatment × session interaction effect (*F*(2,76) = 1.566, *p* = .10, ŋ^2^ = .04), indicating no treatment-specific improvement in attachment anxiety across assessment sessions (see Fig. 2 and Table 2 and for the effect sizes of between-group differences separately for each session). Also no significant within-group pre-to-post changes were identified in the OT or PL group separately (see Table 2).

### Secondary outcome—Inventory of Parent and Peer Attachment

Between-group analyses showed that pre-to-post changes in self-reported secure attachment toward peers and parents were not significantly larger in the OT compared to the PL group (no main effects of treatment: peers: *F*(1,76) = .20, *p* = .33, ŋ^2^ = .01; mother: *F*(1,74) = .57, *p* = .23, ŋ^2^ = .02; father: *F*(1,74) = .78, *p* = .19, ŋ^2^ = .02; nor treatment × session interaction effects: peers: *F*(2,76) = 1.08, *p* = .17, ŋ^2^ = .03; mother: *F*(2,74) = .32, *p* = .36, ŋ^2^ = .01; father: *F*(2,74) = .03, *p* = .49, ŋ^2^ = .00) (see Additional file 1: Figure S2 and Table 2 for the effect sizes of between-group differences separately for each session). Also no significant within-group pre-to-post changes were identified in the OT or PL group separately (see Table 2).

### Secondary outcome—World Health Organization Quality of Life–Bref

Between-group analyses showed that pre-to-post changes in self-reported quality of life were not significantly larger in the OT, compared to the PL group (no main effect of treatment: *F*(1,74) = .77, *p* = .19, ŋ^2^ = .02, nor a treatment x session interaction effect: *F*(2,74) = .96, *p* = .19, ŋ^2^ = .03) (see Additional file 1: Figure S2 and Table 2 for the effect sizes of between-group differences separately for each session). Also, no significant within-group pre-to-post changes were identified in the OT or PL group separately (see Table 2).

### Screening of changes in mood and side effects

As listed in detail in Additional file 1: Table S2, only minimal, non-treatment specific side effects were reported.

In terms of changes in mood as assessed with the Profile of Mood states (POMS), between-group analyses identified a significant main effect of treatment for the mood state “vigor” (*F*(1,76) = 4.09, *p* = .03, ŋ^2^ = .10) (but no treatment × session interaction: *F*(2,76) = .04, *p* = .96, ŋ^2^ = .001), indicating that across assessment sessions, self-reports of “vigor” (feeling “energetic,” “active,” “lively”) were significantly higher in the OT group compared to the PL group (see Table 2 for the effect sizes of between-group differences separately for each session). While no significant pre-to-post changes were evident within the OT group, the PL group showed a significant reduction (compared to baseline) in self-reported vigor at session T1 (*p* = .002) (see Table 2 reporting single-sample *t* tests assessing within-group changes from baseline).

No treatment-specific changes were identified for the other mood states (tension, anger, depression, fatigue) (see Table 2 and Additional file 1: Figure S1), although note that significant reductions (compared to baseline) in feelings of tension and fatigue were reported both in the OT group and in the PL group (see Table 2 reporting single-sample t-tests assessing within-group changes from baseline).

### Associations between ASD characteristics and attachment characteristics

Taken together, treatment-specific effects of a 4-week OT treatment were most pronounced in terms of improvements in repetitive behaviors (RBS-R) and perceived attachment avoidance (SAAM). Here, we specifically explored whether and how the quantitative autism characteristics (SRS-A and RBS-R) (assessed at baseline) were associated with the adopted attachment characteristics (SAAM and IPPA). We additionally explored whether individuals with ASD displayed more impairments in attachment, when their baseline behavioral characterizations were compared to those previously obtained from a sample of neurotypical individuals (*n* = 40, mean age = 21.1, S.D. = 2.6) (data adopted from [17]).

Higher self-reported SRS-A scores (at baseline) (more impairment in social responsiveness) were significantly associated with lower perceived secure attachment (IPPA) towards peers (*r* = − .55, *p* < .001), mother (*r* = − .51, *p* = .001) and father (*r* = − .34, *p* = .034) and with higher perceived attachment avoidance (SAAM) (*r* = .38, *p* = .018), but not with other reports of attachment characteristics (security: *r* = − .26, *p* = .12; anxiety: *r* = .08, *p* = .63). Further, higher scores on the RBS-R (more frequent and/or severe repetitive and restricted behaviors) were significantly associated with lower perceived secure attachment (IPPA) toward the mother (*r* = − .56, *p* < .001), but not the father (*r* = − .31; *p* = .056) or peers (*r* = − .22, *p* = .19). Finally, higher scores on the RBS-R were also significantly associated with higher perceived attachment avoidance (*r* = .50, *p* = .002), but not with other reports of attachment characteristics (security: *r* = − .11, *p* = .50; anxiety: *r* = − .02, *p* = .89).

Notably, exploratory analyses also showed that as a group, the individuals with ASD reported significantly higher perceived attachment avoidance (*t*(76) = − 2.51, *p* = .014), lower attachment security (*t*(76) = 2.48, *p* = .015), and a trend toward lower perceived secure attachment toward peers (IPPA) (*t*(76) = 1.74, *p* = .085), when compared to a neurotypical sample of adult men (data obtained from [17]) (Additional file 1: Table S4).

## Discussion

The current trial demonstrated no treatment-specific effects in the primary outcome assessing social symptoms (SRS-A, self- and informant-rated). In particular, with respect to self-reports of social responsiveness, pre-to-post improvements were evident both in the OT (at session T1 and T2) and in the PL group (at session T2), but with no specific benefit of OT over the PL treatment (no significant between-group difference). Correspondingly, in three previous long-term administration trials, improvements in social symptoms (SRS-A: [9, 12]) (ADOS subscale assessing social reciprocity: 10) were identified both in participants receiving the OT treatment and in those receiving the placebo treatment, but with no significant difference between the two groups. As suggested by Yatawara et al. (2016), one explanation for these unspecific effects may be the presence of a placebo response which has been shown to occur frequently in pediatric autism pharmacological and dietary placebo-controlled trials [37]. In the context of OT trials, increased public attention over the last decade may have influenced the expectations of patients or parents especially with respect to the anticipated effects of OT treatment on social functioning (hence the observed unspecific improvements in self-rated SRS-A scores directly assessing the social domain).

Also with respect to the effect of multiple-dose OT treatment on informant-based reports of social responsiveness (SRS-A), no overall treatment-specific improvements were observed (despite the identification of a reduction in informant-rated symptom severity in the OT group, but not in the PL group at session T2) (see Table 2 reporting within-group changes). In prior studies with young children with ASD, significant treatment-specific improvements in caregiver-rated social responsiveness have been identified immediately after 4 or 5 weeks of OT treatment [13, 14]. The lack of a treatment-specific effect in our trial versus previous trials in children might relate to the frequency of contact of children versus adult populations with their respective informants (i.e., more frequent, sustained child-informant contact), potentially rendering more subtle improvements in social functioning in the adult population to remain undetected by informants.

While no significant treatment-specific effects were identified in social symptoms, exploratory analyses identified long-lasting OT-specific improvements in a secondary outcome assessing attachment characteristics (SAAM), indicating a reduction in feelings of avoidance in the OT, compared to the PL group. While the exact neuromodulatory mechanisms of OT treatment are unknown, OT has been implicated in enhancing the salience of socially relevant cues, inducing reductions in (social) stress and anxiety, and modulating approach/avoidance motivational tendencies, presumably by impacting on limbic circuits (e.g., amygdala) and the central reward system (e.g., nucleus accumbens). As such, by enhancing social salience and reducing social stress/anxiety, the daily OT administrations over a course of 4 weeks, may have induced increased feelings of approachability (reduced avoidance) during social interactions. Furthermore, the observation that these beneficial effects of multiple-dose OT treatment on perceived attachment avoidance outlasted the period of actual administration until 1 month and 1 year post-treatment, provides support to the notion that repeated administrations over an extended period of time might induce long-lasting adaptations in social brain circuits, presumably in an experience-dependent manner. Indeed, through positive re-enforcement, the recursive experience of the social environment as more “secure” or “approachable” (during the period of actual OT administrations) can be anticipated to have contributed to the observed long-lasting adaptations in one’s motivational tendencies (i.e., increased feelings of social approachability). Considering that attachment avoidance reflects a reluctance to trust others and an emphasis on autonomy, whereas attachment anxiety reflects insecurity about oneself (low trust in oneself) and fear of being rejected [33], our results suggest that the 4-week OT treatment predominantly improved a person’s reluctance toward closeness or trust in others (i.e., attachment avoidance), but that it could not induce significant long-term alterations in a person’s feelings of insecurity about one’s own abilities (i.e., attachment anxiety). The notion that OT may thus predominantly influence one’s reluctance to engage in closeness or intimacy with others (rather than one’s fear of being rejected) may be interpreted within the framework of the recently proposed affiliative-motivation hypothesis [38] suggesting that OT specifically acts by increasing affiliative strivings and that individuals with a decreased tendency to affiliate (e.g., avoidantly attached individuals rather than anxiously attached individuals) may be most likely to benefit from OT treatment. Overall, the observation of a beneficial effect of OT on feelings of approachability is in line with findings from a previous study from our lab [17], in which we demonstrated similar treatment-specific reductions in perceived avoidant attachment (as well as improvements in perceived secure attachment to peers) after a 2-week course of OT treatment in neurotypical men. Our findings also extend previous studies showing beneficial effects of a single dose of OT on attachment security [20], development of trust and cooperation [18], and improved communal traits and altered agency [19].

In addition to the treatment-specific effect on attachment avoidance, long-lasting treatment-specific improvements were also identified in terms of repetitive behaviors (RBS-R), indicating an overall reduction in repetitive behaviors in the OT group, compared to the PL group. While the exact link between expressions of repetitive behaviors and difficulties in the social domain is unclear, it has been suggested that at least in a subset of individuals with ASD, the experience of the external (social) milieu as “unapproachable” or even “threatening” may result in an increased “need for sameness” in order to sustain a level of control over the external surroundings [39, 40]. The current study provides preliminary evidence that multiple-dose OT treatment may relieve an individual from this increased “need for sameness” and the resulting need for engaging in repetitive and restricted behaviors. Overall, these observed effects on repetitive behaviors are in line with previous trials with adult men with ASD showing beneficial effects of OT on repetitive behaviors after 4 h of intravenous OT administration [4] and after 6 weeks of daily administrations [8, 10]. Note however that one 1-week trial with adult men with ASD did not show OT-specific improvements on informant-based reports of repetitive behaviors [9]. Also, in previous trials with children and adolescents with ASD, no improvements on repetitive behaviors were observed after 4 days or 4, 5, or 8 weeks of daily OT administration [11–14]. In this view, it appears that beneficial effects of OT treatment on repetitive behaviors were mostly demonstrated in studies that adopted assessments based on self-reports (current study, [4, 8]), whereas no beneficial effects were evident in studies adopting informant-based reports of repetitive behavior [9–14]. Together, these findings may therefore indicate that self-reports, as opposed to informant-based reports, may be more sensitive for capturing subtle, self-experienced changes in repetitive behaviors.

While not included as an explicit outcome measure, screenings of changes in mood states (Profile of Mood States questionnaire (POMS)) showed a treatment-specific effect for the mood state “vigor,” indicating higher reports of feelings of vigor (e.g., feeling “energetic,” “active,” “lively”) in the OT group, compared to the PL group. To our knowledge, this is the first study adopting screenings of mood states in an OT trial with ASD patients. In a previous study from our lab [17], the POMS was also adopted to evaluate the effects of a 2-week OT treatment in neurotypical men, and while here, no treatment-specific changes in vigor were detected, the POMS revealed OT-specific reductions in feelings of tension and anger. In the current study, reductions in feelings of tension were also reported, but irrespective of received treatment, indicating no specific benefit of OT over the PL treatment. While speculative, the stabilizing effect of OT administration on reports of vigor might be related to the (highly understudied, but in the ASD community heavily discussed) phenomenon of “autistic burnout.” Individuals with ASD describe “autistic burnout” as an extreme fatigue and inability to meet the demands of everyday life caused by a continuous attempt to mask and/or deal with their ASD symptoms (i.e., sensory disorders, repetitive behaviors [1]). The overall mitigation of repetitive behavior symptoms and increased feelings of social approachability by the OT treatment may therefore have been accompanied with overall higher reports of feeling “energetic,” “active,” “lively” in the OT group, compared to the PL group. However, considering the exploratory nature of the identified effects, more research is needed to further elucidate the impact of OT treatment on mood states in ASD.

Finally, with respect to the effect of OT on general aspects of quality of life, the current study identified no treatment-specific improvements. To date, evidence on the effects of OT on quality of life is relatively scarce since only two prior studies have addressed this topic. Contrary to our findings, Anagnostou et al. [8] reported an OT-specific improvement in quality of life (socio-emotional section) after a 6-week treatment in adult men with ASD. Watanabe et al. [9] on the other hand, only observed a trend toward improvement in quality of life immediately after a 6-week trial in adult men with ASD. Importantly, recent reviews stated that most individuals with ASD have poor quality of life (note that most studies included children with ASD) [41] or lower quality of life than typically developing adults [42]. Note, however, that to date there is no comprehensive ASD-specific quality of life assessment tool validated and consequently, the tools used in the general population (i.e., WHO-QOL) might not be the most adequate to assess quality of life in ASD (and thus changes in quality of life after intervention) [42].

In terms of associations between core autism characteristics and attachment characteristics, our study showed that impairments in social responsiveness and more frequent and/or severe repetitive behaviors were associated with a more avoidant attachment style and with less secure attachment toward significant others (especially the mother). Albeit exploratory, we also showed that, as a group, the individuals with ASD scored higher on avoidant attachment and lower on secure attachment, when compared to a sample of neurotypical individuals. Together, these findings provide indications that—at least to some extent—associations are evident between core autism characteristics and attachment characteristics, a notion that is generally supported by a recent meta-analysis showing an association between the severity of autism characteristics and less secure attachment in children with ASD [22], as well as by other studies showing more insecure attachment toward parents or romantic partners in unmarried [43] or married adults with ASD [44], respectively. However, since research on this topic to date is limited, it currently remains speculative whether the reported feelings of insecure attachment are a result of the social difficulties experienced by individuals with ASD, or conversely, whether difficulties in the social domain are—in part or within a subset of individuals with ASD—a result of a decreased tendency or inability to form secure attachments. Nevertheless, elucidating the interaction between autism symptomology and attachment style may be of particular relevance in the context of OT treatment, since—according to the aforementioned affiliative-motivation hypothesis—especially individuals with a decreased tendency to affiliate (i.e., avoidantly attached individuals) have been proposed to benefit the most from receiving OT treatment [38]. The current findings of significant ameliorations in attachment avoidance, but no treatment-specific effects on social responsiveness, are in line with this notion and together suggest that attachment characteristics may be more sensitive for evaluating treatment responses, as compared to evaluations based on core autism characteristics alone. Considering the mixed pattern of effects of OT treatment on core autism symptomatology (e.g., SRS-A, RBS-R, ADOS), it seems of great relevance for future multiple-dose clinical trials with individuals with ASD to additionally include more in-depth characterizations of attachment-related constructs both dimensionally and longitudinally (pre-post treatment). In view of the current observations, these explorations are anticipated to be informative for evaluating and predicting treatment responses, and potentially for delineating patient populations that will benefit the most from a course of OT treatment.

### Limitations

Although the current study provides new insights regarding long-lasting effects of multiple-dose OT treatment in ASD and the relation between autism characteristics and attachment characteristics, several limitations need to be considered. First, although our sample size was comparable to that of prior similar clinical trials, studies with larger samples are warranted. Indeed, considering this is an initial pilot study exploring long-term effects of OT treatment (without correction for multiple comparisons), the findings of long-term improvements in repetitive behaviors; attachment avoidance and vigor mood state should be interpreted with caution. Second, in the current study, participants administered the OT nasal spray once a day (in the morning) while the majority of prior multiple-dose OT studies administered two doses/day (one in the morning and one in the afternoon) ([8–10, 12–14], but see [11]). While elevated levels of OT have been demonstrated up to 7 h after a single-dose administration [45], future studies are needed to identify at what point in time the effects of intranasal administration of OT fade out and when OT levels return back to baseline. Also potential interactions with diurnal patterns of endogenous OT levels need to be explored to identify the most optimal dosing and timing of intranasal OT administrations. Third, considering the adopted evaluations were predominantly based on self-report questionnaires, the possibility of subjective bias cannot be ruled out. Participants’ own beliefs about the received treatment, however, were assessed and inclusion of this factor did not modulate the identified treatment effects. Finally, since only adult men with ASD were included, the current observations of beneficial effects of OT treatment cannot be extended to women or children with ASD.

## Conclusions

To conclude, while the 4-week, once-daily treatment with OT induced no treatment-specific changes in primary outcome measures of social symptoms in ASD, exploratory analyses of secondary outcomes showed long-lasting, medium to large-sized improvements in repetitive behaviors and a reduction in perceived attachment avoidance, that outlasted the period of actual administration until 1 month and even 1-year post-treatment. Overall, the observation that the OT treatment primarily targeted long-term adaptations in repetitive behaviors and perceived attachment characteristics indicates that these constructs are sensitive for capturing OT treatment effects in adult men with ASD. In line with the central role of the human oxytocinergic system in interpersonal bonding, trust, and attachment, the current observations may therefore urge future multiple-dose clinical trials to continue to include characterizations of attachment-related constructs when evaluating the potential of OT treatment for ASD. While the exploratory observations of long-term beneficial effects of OT treatment on repetitive behaviors and perceived attachment avoidance are promising, future studies are warranted to further elucidate the long-term impact of OT treatment.

## Supplementary information

**Additional file 1: **Supplementary Methods. Supplementary Results. **Figure S1**. Screening for changes in mood states. **Figure S2**. Effects of oxytocin treatment on attachment and quality of life. **Table S1**. Detailed information on comorbidities and medication use for participants of the oxytocin and placebo treatment groups. **Table S2**. Side effect screening. **Table S3**. Assessment of baseline differences between groups. **Table S4**. Baseline attachment comparison between individuals with ASD and typically developing control subjects (data adopted from (19)) using SAAM and IPPA.

## Data Availability

The datasets used and/or analyzed during the current study are available from the corresponding author on reasonable request.

## Abbreviations

ADOS: Autism Diagnostic Observation Schedule
ASD: Autism spectrum disorder
DSM: Diagnostic and Statistical Manual of Mental Disorders
IPPA: Inventory of Parent and Peer Attachment
IU: International units
MRI: Magnetic resonance imaging
OT: Oxytocin
PL: Placebo
POMS: Profile of Mood States
RBS-R: Repetitive Behavior Scale-Revised
SAAM: State Adult Attachment Measure
SRS-A: Social Responsiveness Scale
WHO-QOL: World Health Organization Quality of Life
