# Supplementary material for: Behavioral effects of multiple-dose oxytocin treatment in autism: a randomized, placebo-controlled trial with long-term follow-up
Source: Mol Autism. 2020 Jan 15;11:6. doi: 10.1186/s13229-020-0313-1 (PMC6964112; doi:10.1186/s13229-020-0313-1)
Supplement: Supplementary file 1 — Additional file 1: Supplementary Methods. Supplementary Results. Figure S1. Screening for changes in mood states. Figure S2. Effects of oxytocin treatment on attachment and quality of life. Table S1. Detailed information on comorbidities and medication use for participants of the oxytocin and placebo treatment groups. Table S2. Side effect screening. Table S3. Assessment of baseline differences between groups. Table S4. Baseline attachment comparison between individuals with ASD and typically developing control subjects (data adopted from (19)) using SAAM and IPPA. [file 13229_2020_313_MOESM1_ESM.pdf]

Supplementary Information

Behavioral effects of multiple-dose oxytocin treatment in autism: A randomized, placebo-controlled trial with long-term follow-up

Sylvie Bernaerts, Bart Boets, Guy Bosmans, Jean Steyaert, Kaat Alaerts

Additional file 1. Supplementary Methods

Detailed questionnaire descriptions.

Social Responsiveness Scale − Adult version (SRS-A)

The SRS-A is a questionnaire developed to measure social functioning in both clinical and nonclinical populations (1). The Dutch-version of the SRS-A consists of 64 items that are rated on a four-point Likert scale ranging from 1 (untrue), 2 (sometimes true), 3 (often true) to 4 (almost always true) (2). Raters were asked to refer to the previous month when completing the scale. The SRS-A encompasses four subscales, including social communication (22 items; α= 0.88), social awareness (19 items; α= 0.80), social motivation (11 items; α= 0.83) and rigidity/repetitiveness (12 items; α= 0.79). SRS-A raw total scores were adopted, for which higher scores indicate lower social responsiveness.

Repetitive Behavior Scale – Revised (RBS-R)

The RBS-R is a questionnaire developed to assess the severity and frequency of restricted and repetitive behaviors (RRBs) observed in ASD (3). The questionnaire consists of 43 items that are rated on a four-point Likert scale ranging from 0 (behavior does not occur), 1 (behavior occurs and is a minor problem), 2 (behavior occurs and is a moderate problem), to 3 (behavior occurs and is a severe problem). Raters were asked to refer to the previous month when completing the scale. The RBS-R encompasses five subscales, including stereotypic behavior (9 items; α= 0.85), self-injurious behavior (8 items; α= 0.84), compulsive behavior (6 items; α= 0.79), ritualistic/sameness behavior (12 items; α= 0.91) and restricted

interests (3 items; α= 0.78) (5 items are not considered in scoring) (4). RBS-R raw total scores were adopted, for which higher scores indicate a higher frequency and/or higher severity of restricted and repetitive behaviors.

State Adult Attachment Measure (SAAM)

The SAAM is a questionnaire designed to assess temporary fluctuations in state attachment (5). For a long time, an adult's attachment style was considered a relatively stable disposition, rooted in internal cognitive–affective working models (i.e., mental representations) of self and other, based on previous experiences in close relationships (6). More recently however, it has been suggested that attachment style can be transiently influenced or shaped by situational factors such as major life events or other contextual factors (7–13). While not contesting the stability of attachment style, the SAAM questionnaire has been validated as a useful measure for capturing temporary fluctuations in the thoughts, feelings, and behaviors underlying attachment processes (5). Although the SAAM is more commonly used to assess attachment priming (e.g. 14–16) or as outcome measure to assess the effect of touch in neurotypicals (e.g. 17,18), prior work from our lab has indicated its relevance for human OT research (19).

The questionnaire contains 21 statements to which participants have to indicate their current state ('right now') on a seven-point Likert scale ranging from 1 (totally disagree), 2 (disagree), 3 (rather disagree), 4 (agree/disagree), 5 (rather agree), 6 (agree), to 7 (totally agree). The SAAM comprises three subscales assessing (i) attachment security (7 items; α= 0.82–0.91); (ii) attachment anxiety (7 items; α= 0.81-0.85); and (iii) attachment avoidance (7 items; α= 0.71-0.87). As a concept, attachment anxiety is characterized to reflect insecurity about one's own worth and abilities, extreme need for interpersonal closeness, love, and support, and worrying about being rejected or abandoned (e.g., "I feel a strong need to be unconditionally loved right now"). Attachment avoidance on the other hand, is characterized by the reluctance to trust others, an emphasis on autonomy and self-reliance, a relatively low tolerance for interpersonal intimacy and interdependence, and a tendency to downregulate one's own emotions (e.g., "If someone tried to get close to me, I would try to keep my

distance"). Reports of attachment security on the other hand, were shown to be inversely related to attachment avoidance and attachment anxiety, which is in line with the conceptualization that attachment security reflects the relative absence of anxiety and avoidance as well as a sense of faith in the responsiveness of attachment figures, and comfort with intimacy and interdependence (e.g., "I feel like I have someone to rely on").

SAAM raw subscale scores were adopted, for which higher scores indicate lower perceived secure attachment on the attachment avoidance and attachment anxiety subscales, and higher perceived secure attachment on the attachment security subscale.

Inventory of Parent and Peer Attachment (IPPA)

The IPPA is a questionnaire designed to assess trait attachment to (i) mother (12 items; α= 0.87); (ii) father (12 items; α= 0.89); (iii) peers (12 items; α= 0.92) (20). More specifically, based on the attachment theory as formulated by Bowlby (6), the IPPA was developed to investigate how well parents and close friends serve as sources of psychological security, by assessing adolescents' perceptions of the positive and negative affective/cognitive dimension of relationships with their parents and peers (degree of mutual trust, quality of communication, and extent of anger and alienation). While commonly used as an instrument for assessing the relation between attachment characteristics and psychopathology (e.g. 22–24), prior work from our lab has indicated its relevance for human OT research (19).

The questionnaire comprises 36 items (12 for each section) with a four-point Likert scale ranging from 1 (almost never), 2 (sometimes), 3 (often), to 4 (almost always). Each section consists of the same 12 items, but with a different person mentioned according to the section (e.g. "I wish I had (a) different mother/father/peers")(20). These items represent the three aforementioned dimensions: trust (e.g. "My mother accepts me as I am"), communication (e.g. "I tell my father about my problems and troubles"), and alienation (e.g. "I feel alone or apart when I am with my friends").

Note that one participant of the OT group did not complete the section on 'father' and another participant of the OT group did not complete the section on 'mother'. IPPA raw subscale

scores were adopted, for which higher scores indicate increased feelings of secure attachment towards peers or parents.

World Health Organization Quality of Life (WHO-QOL) – Bref

The abbreviated version of the WHO-QOL is a 26-item questionnaire with a five-point Likert scale ranging from 1 (not at all, never, very dissatisfied or very poor) to 5 (an extreme amount/extremely, always, very satisfied or very good) (depending on the subscale) to assess general quality of life related to physical health (7 items; $\alpha = 0.82$), psychological health (6 items; $\alpha = 0.75$), social relationships (3 items; $\alpha = 0.66$), and environment (8 items; $\alpha = 0.80$) (2 items are examined separately) (25). WHO-QOL raw total scores were adopted, for which higher scores indicate better quality of life.

Supplementary Results

Exploratory analyses assessing the effect of the participants' own belief about the received treatment on treatment response.

Note that the following secondary analyses are highly exploratory in nature and potentially unreliable, considering that the majority (79.5%) of participants believed that they had received the PL treatment.

		Actual treatment	
		OT	PL
Own belief	OT	6	2
	PL	15	16

Exploratory analyses were performed to assess whether treatment effects on self-report and informant-based social responsiveness (SRS-A), repetitive behaviors (RBS-R), attachment characteristics (SAAM + IPPA) and quality of life (WHO-QOL) were modulated by the participants' own belief about the received treatment. To do so, change-from-baseline scores were subjected to a mixed-effects analysis with the factor 'subject' modeled as random effect and the factors 'treatment group' (OT, PL), 'session' (T1, T2, T3), 'own belief' (OT, PL), and the 'treatment x session' and 'treatment x own belief' interactions modelled as fixed effects.

For none of the outcome measures (except for the IPPA mother subscale) a significant main effect of 'own belief' was revealed (all $p > .05$), indicating that across treatment groups, the participants' own beliefs did not significantly affect the treatment response. For the IPPA mother subscale, a main effect of 'own belief' was found ($F(1,72)= 6.69$, $p= .007$), indicating that across treatment groups, those participants who believe to have received the OT

treatment reported more secure attachment towards their mother compared to those who believed to have received the PL treatment.

For none of the outcomes (except for the SAAM avoidance subscale) a significant 'treatment x own belief' interaction effect was revealed (all $p > .05$). For the SAAM avoidance subscale, a significant 'treatment x own belief' interaction effect was revealed ($F(1,74)= 4.54$, $p= .02$), indicating that in the PL group, participants who believed they had received the OT treatment, reported a greater reduction in perceived attachment avoidance, compared to those who believed to have received the PL treatment (post-hoc Tukey: $p= .02$). Unexpectedly, in the OT group, participants who believed to have received the PL treatment reported a greater reduction in perceived attachment avoidance compared to those who believed to have received the OT treatment (post-hoc Tukey: $p= .03$). However, of those participants who believe to have received the PL treatment, those who actually received the OT treatment did indeed report a greater reduction in perceived attachment avoidance compared to those who had received the PL treatment (post-hoc Tukey: $p= .0002$). Nevertheless, this significant interaction did not change the main effect of treatment ($F(1,74)= 4.20$, $p= .02$), indicating that - across assessment sessions - improvements (from baseline) were significantly larger in the OT, compared to the PL group.

As stated before, considering the majority (79.5%) of participants believed that they had received the PL treatment, these analyses can be unreliable and their interpretation warrants caution.

Supplementary Figures

Figure S1. Screening for changes in mood states.

A 32-item short version of the Profile of Mood States (POMS) questionnaire (26.27) was used as a measure of transient affective states in order to assess whether mood levels of participants changed over the course of the study. This instrument comprises emotional adjectives subdivided in five domains: tension (6 items; α = 0.84). depression (8 items; α = 0.91). vigor (5 items; α = 0.81). fatigue (6 items; α = 0.90) and anger (7 items; α = 0.87) which have to be rated on a five-point Likert scale ranging from 0 (not at all), 1 (a little), 2 (moderately), 3 (quite a lot), to 4 (extremely). Only for the vigor scale, higher scores indicate improvement. Participants were asked to rate their current mood state ('right now'). For all participants, the POMS questionnaire was assessed at baseline, after administration of a single dose of nasal spray (SD); after four consecutive weeks of daily nasal spray administration (T1); and at two follow-up sessions, one month (T2) and one year post-treatment (T3). Also, during the four consecutive weeks of daily nasal spray administration, participants were asked to complete the POMS questionnaire at the end of each week (W1, W2, W3 and W4).

Similar to the main analyses, linear mixed-effects models were constructed (with the random factor 'subject', and the fixed factors 'treatment' (OT, PL), 'session' (T1, T2, T3) and 'treatment x session' interaction) to assess treatment-specific changes in mood states (one-tailed).

For the mood state 'vigor', this analysis revealed a significant main effect of treatment ($F(1,76)= 4.09$, $p= .03$, $\eta^2= .10$) (but no treatment x session interaction: $F(2,76)= .04$, $p= .96$, $\eta^2= .001$), indicating that across assessment sessions (T1, T2, T3), self-reports of 'vigor' (feeling 'energetic', 'active', 'lively') were significantly higher in the OT group compared to the PL group (see **Table 2** for the effect sizes of between-group differences separately for each session). While no significant pre-to-post changes were evident within the OT group, the PL group showed a significant reduction (compared to baseline) in self-reported vigor at session T1 ($p= .002$) (see **Table 2** reporting single-sample t-tests

assessing within-group changes from baseline). No significant effects of treatment (all $F< .33$, $p> .29$) or treatment x session interactions (all $F< 2.05$, $p> .07$) were revealed for the other scales, although note that significant reductions (compared to baseline) in feelings of tension and fatigue were reported both in the OT group and in the PL group (see **Table 2**).

Linear mixed-effects models including the additional assessments of changes in mood states after the single-dose (SD) and during the course of treatment (weekly time points: W1, W2, W3, W4) (with the random factor 'subject', and the fixed factors 'treatment' (OT, PL), 'session' (SD, W1, W2, W3, W4, T1, T2, T3) and 'treatment x session' interaction) revealed a similar main effect of treatment for the mood state 'vigor' (i.e. higher self-reported vigor in the OT compared to the PL group) ($F(1,266)= 3.18$, $p= .04$, $\eta^2= .08$). For the 'depression' scale, a significant treatment x session interaction was revealed ($F(7,266)= 1.80$, $p= .044$, $\eta^2= .05$), indicating a mild-to-medium improvement in feelings of depression in the OT group during the last two weeks of treatment (W3: $d= -.25$; W4: $d= -.48$). No significant main effects of treatment (all $F< .29$, $p> .29$) or treatment x session interactions (all $F< .79$, $p> .30$) were revealed for the other mood states. Vertical bars denote +/- standard errors. Asterisks (*) indicate Cohen's $d \geq .50$ (medium-sized effect).

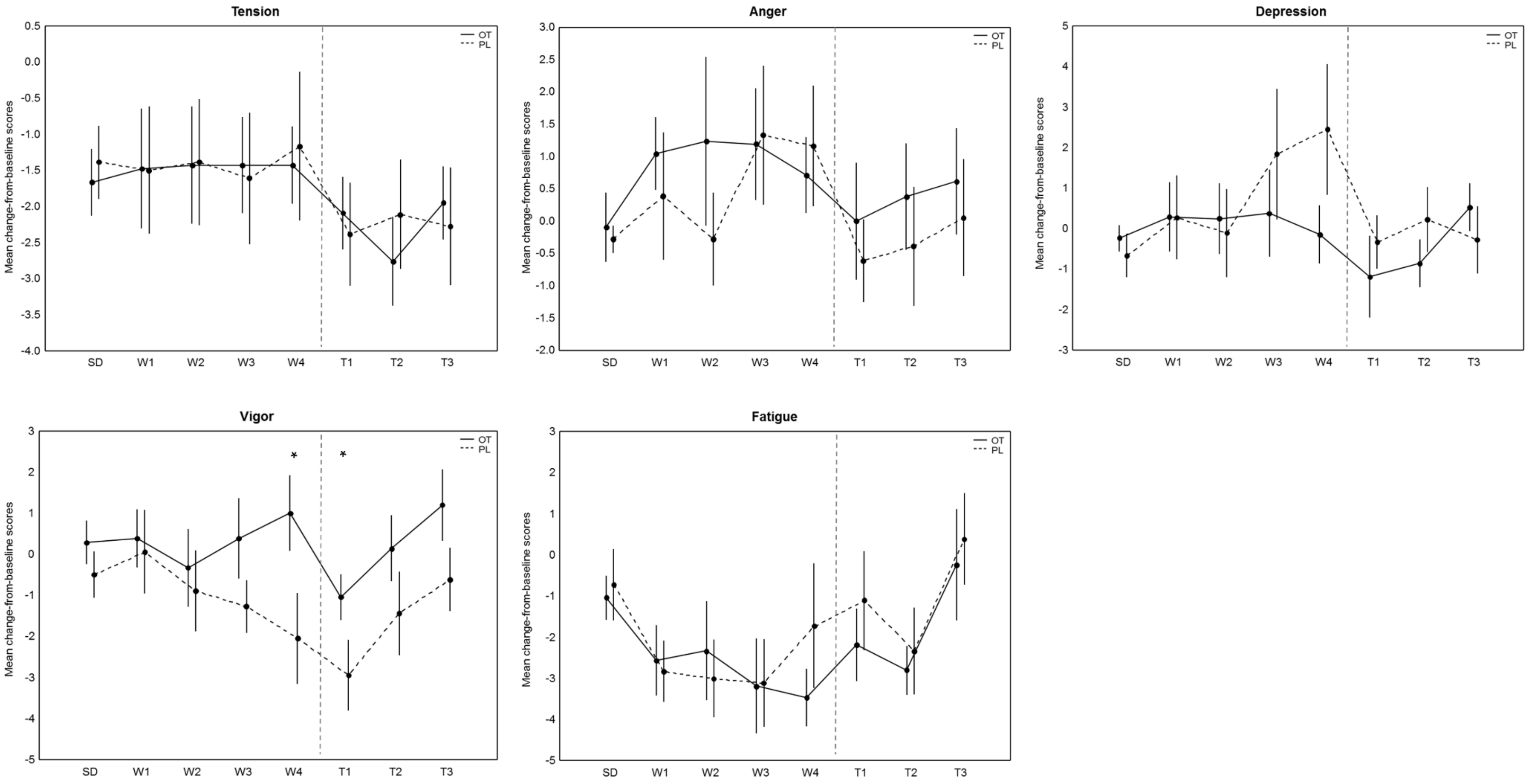
Tension
Anger
Depression
Vigor
Fatigue
OT
PL
Mean change-from-baseline scores
SD
W1
W2
W3
W4
T1
T2
T3
*
*

Figure S2. Effects of oxytocin treatment on attachment and quality of life.

Mean pre-to-post changes (change from baseline) on self-report questionnaires are visualized for the oxytocin (OT) and placebo (PL) treatment groups at assessment session ‘T1’ (immediately after the four-week treatment), ‘T2’ (at follow-up, one month post-treatment) and ‘T3’ (at follow-up, one year post-treatment). Mean changes from baseline are visualized separately for (A) Inventory of Parent and Peer Attachment (IPPA) Peers subscale, (B) IPPA Mother subscale, (C) IPPA Father subscale, and (D) World Health Organization Quality of Life questionnaire (WHO-QOL). Higher scores indicate improvement. Vertical bars denote +/- standard errors.

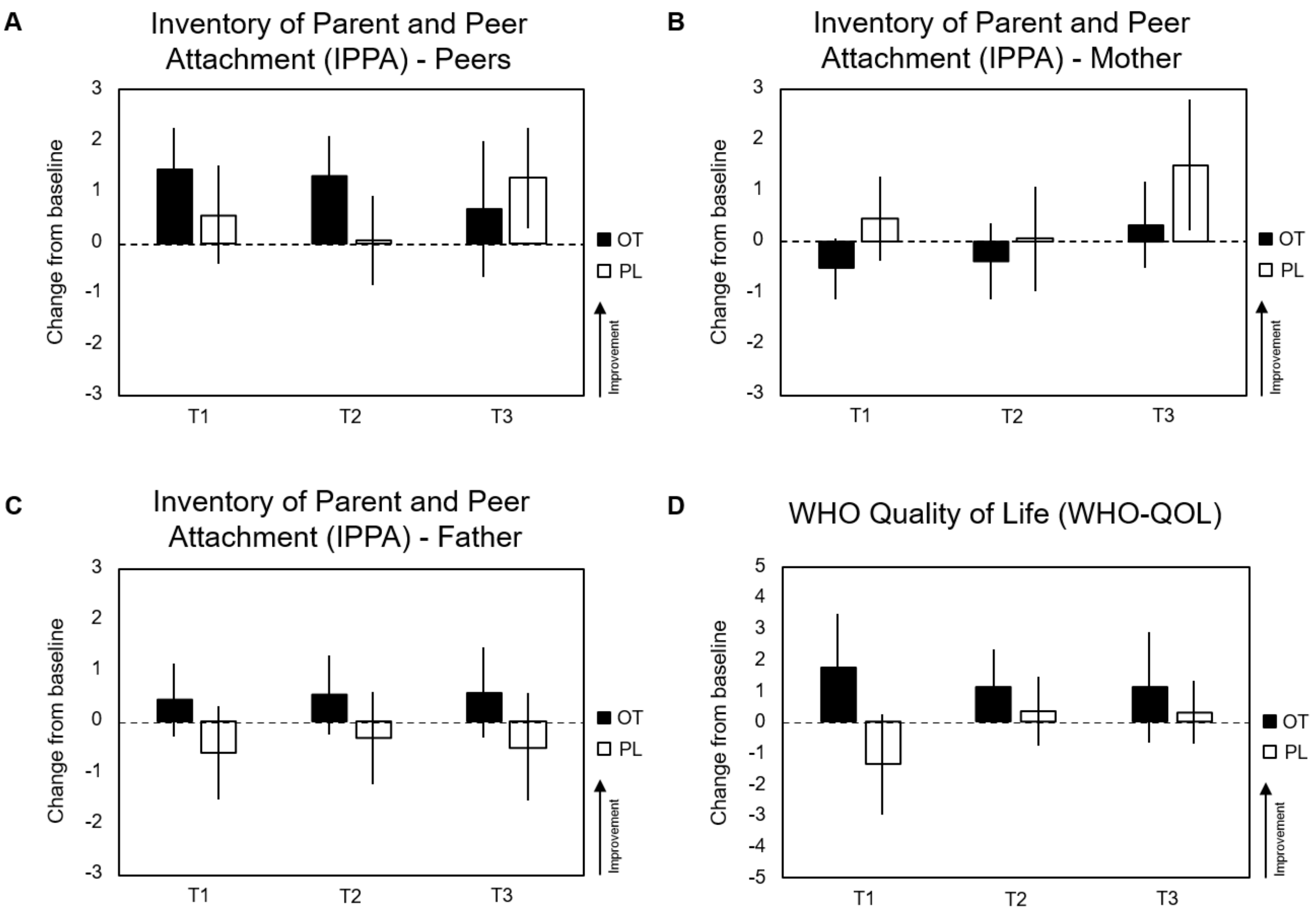

Supplementary Tables

Table S1. Detailed information on comorbidities and medication use for participants of the oxytocin and placebo treatment groups.

Comorbidities were screened through self-report (with the explicit mentioning of examples in the screening interview including e.g., ADHD, depression, dyscalculia, dyslexia). Current psychoactive medication use was defined as use within three months before study enrolment.

		Comorbidities	Medication use
**Oxytocin**			
	**i**	ADHD	Abilify, Tegretol
	**ii**	ADHD, Dyslexia	/
	**iii**	ADHD, Depression	/
	**iv**	ADHD, Bipolar disorder	Antipsychotics (not specified)
	**v**	Depression	Welbutrine XR, Leviron, Cymbalta
	**v**	Depression, ADD	Trazodone Mylan, Medikinet
	**vii**	Bipolar Disorder	Maniprex, Bellozal, Mometasone
	**viii**	Dyslexia	/
	**ix**	/	Risperdal, Venlafaxine
**Placebo**			
	**i**	ADHD	/
	**ii**	ADHD	/
	**iii**	/	Zolpidem, Remergon, Rilatine
	**iv**	/	Trazodone, Escitalopram

ADD = Attention Deficit Disorder, ADHD = Attention Deficit Hyperactivity Disorder.

Table S2. Side effect screening

Participants administered the nasal spray (oxytocin (OT) or placebo (PL)) daily for four consecutive weeks. At the end of each week, participants were asked to report whether they presented any of the listed (or other) side effects and to indicate the severity of the side effect (mild, moderate, or severe). **Panel A** lists the proportion of OT or PL participants (%) that reported any mild, moderate or severe side effects (averaged across effects). **Panel B** lists, separately for each side effect, the proportion of OT or PL participants that reported the side effect (averaged across severity level (mild, moderate, severe)). No significant group differences were revealed in the total proportion of reported side effects (**Panel A**), or separately for each side effect (**Panel B**). Closer inspection showed that a tentatively larger proportion of PL participants reported to feel 'more calm, relaxed, comfortable' (p= .06) after week 2, 3 and 4 of the treatment and 'more confident' (p= .06) after week 4 (indicated in italic in **Panel B**). A marginal effect was also revealed for the side effect 'dry throat/dry mouth' (p= .06), indicating that a tentatively larger proportion of OT participants reported this side effect after week 1 (indicated in italic in **Panel B**). Overall, the most frequent reported side effect was 'runny nose', but the proportion of OT (17.9%) and PL (9.8%) participants reporting this effect was not significantly different (p= .47).

**Panel A**	**OT (%)**			**PL (%)**			**Group Difference (p-value)**		
	Mild	Moderate	Severe	Mild	Moderate	Severe	Mild	Moderate	Severe
**Week 1**	47.6	19.0	9.5	27.8	5.6	5.6	0.21	0.22	0.65
**Week 2**	38.1	9.5	0.0	27.8	11.1	5.6	0.50	0.87	0.28
**Week 3**	23.8	9.5	0.0	33.3	16.7	5.6	0.52	0.51	0.28
**Week 4**	42.9	9.5	0.0	22.2	11.1	5.6	0.18	0.87	0.28
**Across weeks**	38.1	11.9	2.4	27.8	11.1	5.6	0.50	0.94	0.61

**Panel B**	**Week 1**			**Week 2**			**Week 3**			**Week 4**		
	**OT (%)**	**PL (%)**	**p-value**	**OT (%)**	**PL (%)**	**p-value**	**OT (%)**	**PL (%)**	**p-value**	**OT (%)**	**PL (%)**	**p-value**
Headache	4.8	0.0	0.35	0.0	0.0	1.00	0.0	0.0	1.00	0.0	5.6	0.28
Drowsiness	4.8	0.0	0.35	4.8	5.6	0.91	4.8	0.0	0.35	4.8	0.0	0.35
Dizziness	4.8	0.0	0.35	4.8	0.0	0.35	0.0	0.0	1.00	0.0	0.0	1.00
Fainting	0.0	0.0	1.00	0.0	0.0	1.00	0.0	0.0	1.00	0.0	0.0	1.00
Changes in heart rate or palpitations	0.0	0.0	1.00	0.0	0.0	1.00	4.8	0.0	0.35	0.0	0.0	1.00
Shortness of breath	0.0	5.6	0.28	0.0	0.0	1.00	0.0	0.0	1.00	0.0	0.0	1.00
Fever	0.0	0.0	1.00	0.0	0.0	1.00	0.0	0.0	1.00	0.0	0.0	1.00
Sore throat	9.5	0.0	0.19	0.0	0.0	1.00	0.0	0.0	1.00	0.0	0.0	1.00
Dry throat/dry mouth	19.0	0.0	*0.06*	0.0	0.0	1.00	0.0	0.0	1.00	0.0	0.0	1.00
Hoarseness	0.0	0.0	1.00	0.0	0.0	1.00	0.0	0.0	1.00	0.0	0.0	1.00
Coughing	4.8	5.6	0.91	4.8	0.0	0.35	0.0	0.0	1.00	0.0	0.0	1.00
Coughing up mucus	4.8	5.6	0.91	9.5	5.6	0.65	4.8	5.6	0.91	4.8	5.6	0.91
Congested nose	9.5	16.7	0.51	4.8	5.6	0.91	9.5	0.0	0.19	4.8	5.6	0.91
Sneezing	9.5	0.0	0.19	0.0	5.6	0.28	0.0	5.6	0.28	4.8	0.0	0.35
Nasal irritation	4.8	11.1	0.47	4.8	11.1	0.47	4.8	16.7	0.23	9.5	16.7	0.51
Runny nose	23.8	11.1	0.31	14.3	5.6	0.38	23.8	5.6	0.13	9.5	16.7	0.51
Burning sensation in nose and/or ears	9.5	5.6	0.65	4.8	0.0	0.35	0.0	5.6	0.28	0.0	5.6	0.28
Sensitive to fragrances	0.0	0.0	1.00	0.0	0.0	1.00	0.0	0.0	1.00	0.0	0.0	1.00
Watery eyes	0.0	0.0	1.00	0.0	0.0	1.00	0.0	0.0	1.00	0.0	0.0	1.00
Nausea and/or vomiting	4.8	0.0	0.35	0.0	0.0	1.00	0.0	0.0	1.00	0.0	0.0	1.00
Abdominal or stomach pain	4.8	0.0	0.35	0.0	0.0	1.00	0.0	0.0	1.00	0.0	0.0	1.00
Decreased appetite	9.5	0.0	0.19	4.8	0.0	0.35	0.0	0.0	1.00	0.0	0.0	1.00
Hungry	0.0	5.6	0.28	0.0	0.0	1.00	0.0	0.0	1.00	0.0	5.6	0.28
Constipation	0.0	0.0	1.00	0.0	0.0	1.00	0.0	0.0	1.00	0.0	0.0	1.00
Diarrhea	0.0	0.0	1.00	0.0	0.0	1.00	0.0	0.0	1.00	0.0	0.0	1.00
Muscle pain/cramps	0.0	0.0	1.00	0.0	0.0	1.00	0.0	0.0	1.00	0.0	0.0	1.00

Skin rash	0.0	0.0	1.00	0.0	0.0	1.00	0.0	5.6	0.28	0.0	0.0	1.00
Increased fluid intake	0.0	5.6	0.28	0.0	0.0	1.00	0.0	0.0	1.00	0.0	0.0	1.00
Water retention/bloating	0.0	0.0	1.00	0.0	0.0	1.00	0.0	0.0	1.00	0.0	0.0	1.00
Insomnia/sleep difficult	0.0	5.6	0.28	0.0	0.0	1.00	0.0	0.0	1.00	4.8	0.0	0.35
Nightmares	0.0	0.0	1.00	0.0	0.0	1.00	0.0	0.0	1.00	0.0	0.0	1.00
Staring/daydreams	4.8	0.0	0.35	4.8	0.0	0.35	0.0	0.0	1.00	0.0	0.0	1.00
Anaphylaxis	0.0	0.0	1.00	0.0	0.0	1.00	0.0	0.0	1.00	0.0	0.0	1.00
Changes in perception of the tongue	0.0	0.0	1.00	0.0	0.0	1.00	0.0	0.0	1.00	0.0	0.0	1.00
Back pain	0.0	0.0	1.00	0.0	0.0	1.00	0.0	0.0	1.00	0.0	0.0	1.00
Bed wetting	0.0	0.0	1.00	0.0	0.0	1.00	0.0	0.0	1.00	0.0	0.0	1.00
Weight gain	0.0	0.0	1.00	4.8	0.0	0.35	0.0	0.0	1.00	0.0	5.6	0.28
Sweating	0.0	0.0	1.00	0.0	0.0	1.00	0.0	0.0	1.00	0.0	0.0	1.00
Blurred vision	0.0	0.0	1.00	0.0	0.0	1.00	0.0	0.0	1.00	0.0	0.0	1.00
Less talk to others	0.0	0.0	1.00	4.8	0.0	0.35	0.0	0.0	1.00	0.0	0.0	1.00
Uninterested in others	0.0	0.0	1.00	0.0	5.6	0.28	0.0	0.0	1.00	0.0	0.0	1.00
Persistent thoughts and/or feelings	4.8	0.0	0.35	0.0	0.0	1.00	0.0	0.0	1.00	0.0	0.0	1.00
Development of repetitive behavior	0.0	0.0	1.00	0.0	0.0	1.00	0.0	0.0	1.00	0.0	0.0	1.00
Increase in repetitive behavior	0.0	0.0	1.00	0.0	0.0	1.00	0.0	0.0	1.00	0.0	0.0	1.00
Nail biting	0.0	0.0	1.00	0.0	0.0	1.00	0.0	0.0	1.00	0.0	0.0	1.00
Annoyed. bored	4.8	0.0	0.35	0.0	0.0	1.00	0.0	0.0	1.00	0.0	0.0	1.00
Sad	0.0	0.0	1.00	0.0	0.0	1.00	0.0	0.0	1.00	0.0	0.0	1.00
Prone to crying. more emotional	0.0	0.0	1.00	0.0	0.0	1.00	4.8	0.0	0.35	4.8	0.0	0.35
Anxious. worried. discomfort	0.0	5.6	0.28	0.0	5.6	0.28	0.0	11.1	0.13	0.0	5.6	0.28
Happy. satisfied	4.8	11.1	0.47	4.8	16.7	0.23	4.8	11.1	0.47	4.8	11.1	0.47
Euphoric. unusually happy.	0.0	11.1	0.13	0.0	11.1	0.13	0.0	5.6	0.28	4.8	5.6	0.91
Calm. relaxed. comfortable	4.8	11.1	0.47	0.0	16.7	*0.06*	0.0	16.7	*0.06*	0.0	16.7	*0.06*
More focused	0.0	11.1	0.13	0.0	5.6	0.28	0.0	11.1	0.13	0.0	11.1	0.13
More confidence	0.0	11.1	0.13	0.0	11.1	0.13	0.0	11.1	0.13	0.0	16.7	*0.06*

Table S3. Assessment of baseline differences between groups.

**Outcome measure**	**Oxytocin**		**Placebo**			
	**N**	**Mean ± SD**	**N**	**Mean ± SD**	**T-value**	**p-value**
SRS-A self-report	22	72.82 ± 30.37	18	66.89 ± 23.95	0.67	0.50
SRS-A Informant-based	17	78.71 ± 30.43	15	71.47 ± 29.67	0.68	0.50
RBS-R	22	15.59 ± 16.16	17	8.82 ± 8.41	1.57	0.13
SAAM Avoidance	22	3.09 ± 1.26	18	2.90 ± 0.93	0.54	0.59
SAAM Security	22	5.27 ± 0.70	18	5.31 ± 0.82	-0.18	0.86
SAAM Anxiety	22	4.05 ± 1.11	18	3.83 ± 0.95	0.66	0.51
IPPA Peers	22	33.14 ± 6.68	18	33.89 ± 6.29	-0.36	0.72
IPPA Mother	21	36.33 ± 8.36	18	37.22 ± 5.40	-0.39	0.70
IPPA Father	21	31.57 ± 9.93	18	33.94 ± 9.03	-0.78	0.44
WHO-QOL	22	82.91 ± 14.04	17	85.24 ± 9.63	-0.58	0.56

SRS-A = Social Responsiveness Scale adult version, RBS-R = Repetitive Behavior Scale – Revised, SAAM = State Adult Attachment Measure, IPPA = Inventory of Parent and Peer Attachment, WHO-QOL = World Health Organization Quality of Life questionnaire.

Table S4. Baseline attachment comparison between individuals with ASD and typically developing control subjects (data adopted from (19)) using SAAM and IPPA.

	ASD (N = 38)	CON (N = 40)	T-value	p-value
**SAAM**				
Avoidance	3.01 ± 1.14	2.37 ± 1.12	-2.51	0.01
Security	5.30 ± 0.76	5.73 ± 0.75	2.48	0.02
Anxiety	3.90 ± 1.02	3.89 ± 0.91	-0.03	0.98
**IPPA**				
Peers	33.47 ± 6.52	35.98 ± 6.15	1.74	0.09
Mother	36.78 ± 7.02	37.28 ± 7.07	0.31	0.76
Father	32.24 ± 9.51	33.33 ± 7.20	0.57	0.57

All data are shown as mean ± standard deviation. ASD = Autism Spectrum Disorder. CON = typically developing control subjects. SAAM = State Adult Attachment Measure. IPPA = Inventory of Parent and Peer Attachment.

References

1. Constantino JN. Social Responsiveness Scale-Adult Research Version. Lon Angeles. CA: Western Psychological Services; 2005.

2. Noens I. De La Marche W. Scholte E. SRS-A: Screeningslijst voor autismespectrumstoornissen. Handleiding. Amsterdam. The Netherlands: Hogrefe Uitgevers; 2012. 55 p.

3. Bodfish JW. Symons FJ. Parker DE. Lewis MH. Varieties of Repetitive Behavior in Autism: Comparisons to Mental Retardation. J Autism Dev Disord [Internet]. 2000 [cited 2018 Jun 1];30(3):237–43. Available from: http://link.springer.com/10.1023/A:1005596502855

4. Lam KSL. Aman MG. The repetitive behavior scale-revised: Independent validation in individuals with autism spectrum disorders. J Autism Dev Disord. 2007;37(5):855–66.

5. Gillath O. Hart J. Noftle EE. Stockdale GD. Development and validation of a state adult attachment measure (SAAM). J Res Pers [Internet]. 2009 Jun [cited 2016 Feb 15];43(3):362–73. Available from: http://www.sciencedirect.com/science/article/pii/S0092656608001694

6. Bowlby J. Attachment and loss:Volume I. Attachement.

7. Cozzarelli C. Karafa JA. Collins NL. Tagler MJ. Stability and change in adult attachment styles: associations with personal vulnerabilities. life events. and global construals of self and others. J Soc Clin Psychol [Internet]. 2003 Jul [cited 2016 Jun 13];22(3):315–46. Available from: http://guilfordjournals.com/doi/abs/10.1521/jscp.22.3.315.22888

8. Davila J. Burge D. Hammen C. Why Does Attachment Style Change? J Pers Soc Psychol. 1997;73(4):826–38.

9. Davila J. Sargent E. The Meaning of Life (Events) Predicts Changes in Attachment Security. Personal Soc Psychol Bull [Internet]. 2003 Nov 1 [cited 2016 Jun 13];29(11):1383–95. Available from: http://psp.sagepub.com/cgi/doi/10.1177/0146167203256374

10. Feeney JA. Noller P. Attachment style and romantic love: Relationship dissolution. Aust J Psychol. 1992;44(2):69–74.

11. Gillath O. Shaver PR. Effects of attachment style and relationship context on selection among relational strategies. J Res Pers [Internet]. 2007 Aug [cited 2016 Jun 13];41(4):968–76. Available from: http://linkinghub.elsevier.com/retrieve/pii/S009265660600136X

12. Hammond JR. Fletcher GJO. Attachment Styles and Relationship Satisfaction in the Development of Close Relationships. NZ J Psychol. 1991;20:56–62.

13. Bosmans G. Van de Walle M. Goossens L. Ceulemans E. (In)variability of Attachment in Middle Childhood: Secure Base Script Evidence in Diary Data. Behav Chang [Internet]. 2014 Dec 20 [cited 2019 Feb 19];31(04):225–42. Available from: https://www.cambridge.org/core/product/identifier/S0813483914000187/type/journal_article

14. Oehler M. Psouni E. “Partner in Prime”? Effects of repeated mobile security priming on attachment security and perceived stress in daily life. Attach Hum Dev [Internet]. 2018 Sep 20 [cited 2019 Feb 19];1–20. Available from: https://www.tandfonline.com/doi/full/10.1080/14616734.2018.1517811

15. McGuire A. Gillath O. Jackson Y. Ingram R. Attachment Security Priming as a Potential Intervention for Depressive Symptoms. J Soc Clin Psychol [Internet]. 2018 Jan 8 [cited 2019 Feb 19];37(1):44–68. Available from: https://guilfordjournals.com/doi/10.1521/jscp.2018.37.1.44

16. Pan Y. Zhang D. Liu Y. Ran G. Teng Z. The effects of attachment style and security priming on the perception of others' pain. J Soc Pers Relat [Internet]. 2017 Mar 9 [cited 2019 Feb 19];34(2):184–208. Available from: http://journals.sagepub.com/doi/10.1177/0265407515627509

17. Maratos FA. Duarte J. Barnes C. McEwan K. Sheffield D. Gilbert P. The physiological and emotional effects of touch: Assessing a hand-massage intervention with high self-critics. Psychiatry Res [Internet]. 2017 Apr 1 [cited 2019 Feb 19];250:221–7. Available from: https://www.sciencedirect.com/science/article/pii/S0165178116306059?via%3Dihub

18. Jakubiak BK. Feeney BC. A Sense of Security: Touch Promotes State Attachment Security. Soc Psychol Personal Sci [Internet]. 2016 Sep 8 [cited 2019 Feb 19];7(7):745–53. Available from: http://journals.sagepub.com/doi/10.1177/1948550616646427

19. Bernaerts S. Prinsen J. Berra E. Bosmans G. Steyaert J. Alaerts K. Long-term oxytocin administration enhances the experience of attachment. Psychoneuroendocrinology. 2017;78:1–9.

20. Armsden GC. Greenberg MT. The inventory of parent and peer attachment: Individual differences and their relationship to psychological well-being in adolescence. J Youth Adolesc [Internet]. 1987;16(5):427–54. Available from: http://dx.doi.org/10.1007/BF02202939

21. Viana AG. Rabian B. Perceived attachment: Relations to anxiety sensitivity. worry. and GAD symptoms. Behav Res Ther [Internet]. 2008 Jun 1 [cited 2019 Feb 19];46(6):737–47. Available from: https://www.sciencedirect.com/science/article/pii/S0005796708000557?via%3Dihub

22. Smith M. Calam R. Bolton C. Psychological Factors Linked to Self-Reported Depression Symptoms in Late Adolescence. Behav Cogn Psychother [Internet]. 2009

Jan 30 [cited 2019 Feb 19];37(01):73. Available from: http://www.journals.cambridge.org/abstract_S1352465808004724

23. Yates TM. Tracy AJ. Luthar SS. Nonsuicidal self-injury among "privileged" youths: longitudinal and cross-sectional approaches to developmental process. J Consult Clin Psychol [Internet]. 2008 Feb [cited 2019 Feb 19];76(1):52–62. Available from: http://www.ncbi.nlm.nih.gov/pubmed/18229983

24. Prinsen J. Brams S. Alaerts K. To mirror or not to mirror upon mutual gaze. oxytocin can pave the way: A cross-over randomized placebo-controlled trial. Psychoneuroendocrinology [Internet]. 2018 Apr 1 [cited 2018 Jun 21];90:148–56. Available from: https://www.sciencedirect.com/science/article/pii/S0306453018300854?via%3Dihub

25. The Whoqol Group. The World Health Organization quality of life assessment (WHOQOL): Development and general psychometric properties. Soc Sci Med [Internet]. 1998 Jun 15 [cited 2018 Jun 13];46(12):1569–85. Available from: https://www.sciencedirect.com/science/article/pii/S0277953698000094?via%3Dihub

26. McNair DM. Lorr M. An analysis of mood in neurotics. J Abnorm Soc Psychol [Internet]. 1964 [cited 2016 Jun 13];69(6):620–7. Available from: http://doi.apa.org/getdoi.cfm?doi=10.1037/h0040902

27. Wald FD. Mellenbergh GJ. The shortened version of the Dutch translation of the Profile of Mood States (POMS). Ned Tijdschr Psychol. 1990;45(2):86–90.
